# Prevalence of somatic-mental multimorbidity and its prospective association with disability among older adults in China

**DOI:** 10.18632/aging.103070

**Published:** 2020-04-25

**Authors:** Haibin Li, Anxin Wang, Qi Gao, Xiaonan Wang, Yanxia Luo, Xinghua Yang, Xia Li, Wei Wang, Deqiang Zheng, Xiuhua Guo

**Affiliations:** 1 Department of Epidemiology and Health Statistics, School of Public Health, Capital Medical University, Beijing, China; 2 Beijing Municipal Key Laboratory of Clinical Epidemiology, Capital Medical University, Beijing, China; 3 Department of Neurosurgery, Beijing Tiantan Hospital, Capital Medical University, Beijing, China; 4 Department of Mathematics and Statistics, La Trobe University, Melbourne, Victoria, Australia; 5 Global Health and Genomics, School of Medical Sciences and Health, Edith Cowan University, Perth, Western Australia, Australia

**Keywords:** multimorbidity, somatic conditions, mental conditions, disability

## Abstract

We aimed to identify prevalent somatic-mental multimorbidity (SMM) and examine its prospective association with disability among a nationally representative sample. A total of 6728 participants aged 60 years and older in the China Health and Retirement Longitudinal Study were included. A total of 14 somatic or mental conditions were assessed in 2013. SMM was defined as any combination of two or more conditions in which at least one condition was somatic and at least one condition was mental. Disability risk was measured using the combined Activities of Daily Living (ADL)-Instrumental Activities of Daily Living (IADL) index (range 0–11; higher index indicates higher disability) in 2013 and 2015. Overall, the prevalence of SMM was 35.7% (95% confidence interval (CI): 34.1%-37.3%) in 2013. After adjustment for sociodemographic characteristics, lifestyles and baseline ADL-IADL index, over a maximum follow-up period of 2 years, SMM was associated with a 2.61 (95% CI: 2.12-3.22)-fold increase in ADL-IADL disability risk compared with that of healthy participants. In conclusion, SMM was prevalent in older Chinese adults, and it was associated with a higher risk of prospective disability.

## INTRODUCTION

China, a large middle-income country, has the world’s largest aging population [1, 2]. It is estimated that the number of Chinese people aged 65 years or older will reach 400 million by 2050, 150 million of whom will be aged 80 years or older [3]. Currently, stroke, ischemic heart disease, and chronic obstructive pulmonary disease are the top three causes of years of life lost in China [4]. Somatic multimorbidity (defined as two or more chronic diseases [5]) is common and prevalent among older Chinese adults. The approximate prevalence of multimorbidity has been reported to be between 10% and 90% in different study populations in China [6–9]. Numerous epidemiologic studies have reported that multimorbidity is associated with reduced quality of life [10], compromised self-rated health [11], depressive symptoms [12], cognitive impairment [13], disability [14], and all-cause mortality [15]. In addition, multimorbidity leads to more complex economic and healthcare challenges [6].

A growing number of studies have found that mental health conditions such as depression or anxiety are prevalent and commonly co-occur with chronic somatic conditions among the elderly [14, 16–21]. This phenomenon is termed somatic-mental multimorbidity (SMM). Among the elderly, cognitive impairment and depression are major mental conditions [22, 23]. A recent meta-analysis reported that nearly one-third of participants with cognitive impairment suffered from depression [24]. The prevalence of SMM was estimated to be 13.9% in men and 21.1% in women in 2010 among 138,858 American residents aged 65 years or older [20]. To the best of our knowledge, little is known about the prevalence and pattern of combinations of somatic and mental conditions in developing countries, particularly in China.

Several previous studies have demonstrated that somatic multimorbidity increases the risk of disability among the elderly [14, 19, 21, 25–29]. However, the impact of SMM on disability has been less well described [14, 21]. Recently, Vetrano et al. reported that the co-occurrence of cardiovascular and neuropsychiatric chronic diseases increased disability over 9 years of follow-up [21]. In addition, Quinones et al. investigated the impact of different combinations of SMM on disability using a nationally representative prospective cohort study [14]. However, those studies were conducted in Western populations [14, 21]. Little is known about the pattern of SMM and its impact on prospective disability in the Chinese population, which has a higher prevalence of somatic multimorbidity.

The aim of this study is to (1) estimate the prevalence and patterns of SMM by age, sex, residence, and geographical region in a nationally representative Chinese population and (2) explore the association of different combinations of somatic and mental conditions on prospective disability in older adults.

## RESULTS

### Sample characteristics

A total of 6728 older adults aged 60 years or older at baseline was included for the current analysis. Table 1 presents the characteristics of the sample. The mean age was 67.6 ± 6.3 years, and 50.3% of the participants were male. Overall, 50.9% of the participants had somatic multimorbidity, and the prevalence of arthritis was highest (39.9%), followed by hypertension (35.9%). In addition, 33.8% of participants were depressed, and 19.2% had cognitive impairment. Of the participants, 8.4% had both depression and cognitive impairment. At baseline, 39.7% of participants had difficulty in at least one ADL-IADL impairment.

**Table 1:** Baseline characteristics of china health and retirement longitudinal study (Unweighted).

**Variable**	**Value (n=6728)**
Age-years-Mean ± *SD*	67.6 ± 6.3
Male, n (%)	3383 (50.3)
Less than lower secondary education, n (%)	6292 (93.5)
Married, n (%)	5231 (77.8)
Rural area, n (%)	4256 (63.3)
Geographical region, n (%)	
East	2142 (31.8)
North	745 (11.1)
North-East	446 (6.6)
North-West	466 (6.9)
South-Central	1592 (23.7)
South-West	1337 (19.9)
Current smoker, n (%)	936 (13.9)
Current drinker, n (%)	2179 (32.4)
Hypertension, n (%)	2417 (35.9)
Diabetes, n (%)	616 (9.2)
Cancer, n (%)	72 (1.1)
Lung disease, n (%)	1008 (15.0)
Heart disease, n (%)	1176 (17.5)
Stroke, n (%)	260 (3.9)
Arthritis, n (%)	2683 (39.9)
Dyslipidemia, n (%)	916 (13.6)
Liver disease, n (%)	316 (4.7)
Kidney disease, n (%)	509 (7.6)
Stomach disease, n (%)	1719 (25.6)
Asthma, n (%)	459 (6.8)
No. of somatic conditions, n (%)	
0	1437 (21.4)
1	1868 (27.8)
2+	3423 (50.9)
Depression, n (%)	2274 (33.8)
Cognitive impairment, n (%)	1291 (19.2)
No. of mental conditions, n (%)	
0	3730 (55.4)
1	2431 (36.1)
2	567 (8.4)
ADL–IADL index in 2013-Mean ± *SD*	1.0 ± 1.8
1+, n (%)	2667 (39.7)

### Prevalence of SMM

**Figure 1:**
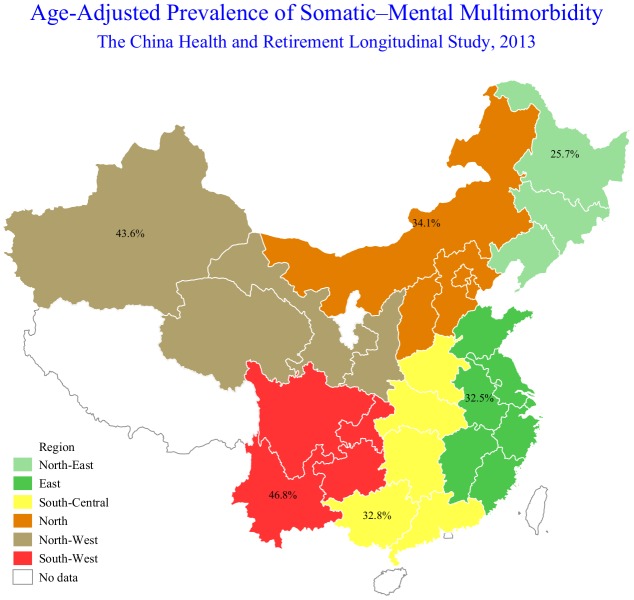
**Flowchart of selection of study participants.**

Table 2 summarizes the sex-specific prevalence of SMM by age and residence. The overall prevalence of SMM was 35.7% (95% confidence interval (CI): 34.1%-37.3%) in China in 2013. Overall, females had a higher prevalence of SMM than males (44.6% vs. 27.0%, *p*-value <0.001), and this difference was significant across different age groups and residence. Among males, the prevalence of SMM was highest in the 70-74 age group compared to those aged 60-64 years (32.8% vs. 24.6%, *p*-value=0.001), while the prevalence of SMM was highest in those aged 75 years or older compared to those aged 60-64 years among females (47.6% vs. 41.6%, *p*-value=0.039). This difference was more obvious when participants were stratified by residence. The SMM prevalence was also higher among one who lived in rural areas than among persons who lived in urban areas in both males (31.9% vs. 20.8%, *p*-value <0.001) and females (52.9% vs. 34.0%, *p*-value <0.001). Figure 1 shows the age-adjusted prevalence of SMM by geographic location in China. The Southwest region (46.8%) had the highest prevalence of SMM, followed by the Northwest (43.6%), North (34.1%), South-Central (32.8%), and East region (32.5%), while participants who lived in the Northeast region (25.7%) had the lowest SMM prevalence.

**Table 2:** Prevalence (per 100 people) of somatic–mental multimorbidity by age-sex-and urban/rural residence.

**Age (y)**	**Male**				**Female**		
	**Total population**	**Prevalence**			**Total Population**	**Prevalence**	
		**Unweighted, n (%)**	Weighted*, % (95% CI)			**Unweighted, n (%)**	Weighted*, % (95% CI)
All urban/rural residence							
60-64	1354	350 (25.9)	24.6 (22.0-27.3)		1339	574 (42.9)	41.6 (38.5-44.8)
65-69	895	247 (27.6)	25.3 (21.6-29.3)		907	429 (47.3)	46.1 (41.1-51.1)
70-74	609	199 (32.7)	32.8 (28.7-37.2)		566	275 (48.6)	45.6 (40.9-50.3)
75+	525	163 (31.1)	29.0 (23.4-35.3)		533	264 (49.5)	47.6 (42.5-52.7)
All ages	3383	959 (28.4)	27.0 (24.9-29.2)		3345	1542 (46.1)	44.6 (42.2-47.0)
Urban residence							
60-64	492	96 (19.5)	18.3 (14.7-22.5)		495	166 (33.5)	31.7 (27.0-37.0)
65-69	310	73 (23.6)	19.6 (14.1-26.5)		301	106 (35.2)	36.4 (25.6-48.6)
70-74	226	60 (26.6)	29.1 (22.6-36.5)		225	75 (33.3)	32.1 (25.4-39.7)
75+	197	47 (23.9)	20.0 (12.6-30.3)		226	91 (40.2)	36.9 (29.8-44.8)
All ages	1225	276 (22.5)	20.8 (17.7-24.3)		1247	438 (35.1)	34.0 (29.8-38.4)
Rural residence							
60-64	862	254 (29.5)	29.2 (25.7-32.9)		844	166 (33.5)	48.9 (44.9-52.9)
65-69	585	174 (29.7)	29.7 (25.8-33.9)		606	106 (35.2)	52.8 (48.4-57.1)
70-74	383	139 (36.3)	35.7 (30.5-41.3)		341	75 (33.3)	57.7 (51.9-63.4)
75+	328	116 (35.4)	37.9 (32.1-44.0)		307	91 (40.2)	57.7 (51.0-64.1)
All ages	2158	683 (31.7)	31.9 (29.4-34.5)		2098	438 (35.1)	52.9 (50.2-55.5)

*Accounted for the complex survey design.

### SMM and prospective disability

Table 3 reports the association between multimorbidity combinations and the ADL-IADL index in 2015. Over a maximum follow-up period of 2 years, SMM was associated with a 4.49 (95% CI: 3.60-5.59)-fold increase in ADL-IADL risk in the unadjusted model and a 2.61 (95% CI: 2.12-3.22)-fold increase in ADL-IADL in the adjusted model compared with healthy participants.

**Table 3:** Association between combination of somatic and mental conditions and ADL–IADL index.

**Multimorbidity combination groups**	**Unadjusted e^β^ (95% CI)**	***p* value**	Adjusted* e^β^ (95% CI)	***p* value**
Total population (n=6728)				
No somatic and mental conditions (n=940)	*Reference*		*Reference*	
Only 1 somatic condition (n=1103)	1.27 (0.96-1.67)	0.089	1.24 (0.97-1.60)	0.091
Somatic conditions multimorbidity (n=1687)	1.78 (1.39-2.27)	<0.001	1.59 (1.28-1.97)	<0.001
Only 1 mental condition (n=413)	2.54 (1.92-3.34)	<0.001	1.88 (1.46-2.41)	<0.001
Mental conditions multimorbidity (n=84)	4.05 (2.75-5.96)	<0.001	2.09 (1.43-3.05)	<0.001
Somatic-mental multimorbidity (n=2501)	4.49 (3.60-5.59)	<0.001	2.61 (2.12-3.22)	<0.001
Age 60-69 y (n=4495)				
No somatic and mental conditions (n=678)	*Reference*		*Reference*	
Only 1 somatic condition (n=783)	1.51 (1.07-2.11)	0.017	1.35 (0.96-1.88)	0.081
Somatic conditions multimorbidity (n=1152)	2.01 (1.48-2.72)	<0.001	1.75 (1.33-2.32)	<0.001
Only 1 mental condition (n=236)	2.70 (1.85-3.95)	<0.001	2.20 (1.52-3.18)	<0.001
Mental conditions multimorbidity (n=46)	4.09 (2.39-7.01)	<0.001	2.35 (1.31-4.22)	0.004
Somatic-mental multimorbidity (n=1600)	5.14 (3.91-6.76)	<0.001	3.05 (2.29-4.07)	<0.001
Age 70+ y (n=2233)				
No somatic and mental conditions (n=262)	*Reference*		*Reference*	
Only 1 somatic condition (n=320)	1.03 (0.67-1.58)	0.896	1.05 (0.71-1.54)	0.811
Somatic conditions multimorbidity (n=535)	1.44 (0.98-2.12)	0.064	1.36 (0.99-1.86)	0.060
Only 1 mental condition (n=177)	1.91 (1.27-2.89)	0.002	1.61 (1.12-2.32)	0.010
Mental conditions multimorbidity (n=38)	3.16 (1.92-5.19)	<0.001	1.94 (1.23- 3.05)	0.004
Somatic-mental multimorbidity (n=901)	3.44 (2.44-4.86)	<0.001	2.10 (1.55-2.85)	<0.001
Male (n=3383)				
No somatic and mental conditions (n=590)	*Reference*		*Reference*	
Only 1 somatic condition (n=665)	1.64 (1.16-2.31)	0.005	1.42 (1.00-2.02)	0.047
Somatic conditions multimorbidity (n=944)	1.99 (1.44-2.75)	<0.001	1.63 (1.20-2.20)	0.002
Only 1 mental condition (n=200)	3.03 (2.10-4.36)	<0.001	2.28 (1.56-3.32)	<0.001
Mental conditions multimorbidity (n=25)	3.53 (1.89-6.60)	<0.001	1.94 (1.09-3.43)	0.024
Somatic-mental multimorbidity (n=959)	5.59 (4.25-7.36)	<0.001	2.84 (2.15-3.75)	<0.001
Female (n=3345)				
No somatic and mental conditions (n=350)	*Reference*		*Reference*	
Only 1 somatic condition (n=438)	0.98 (0.67-1.43)	0.901	1.04 (0.75-1.45)	0.792
Somatic conditions multimorbidity (n=743)	1.58 (1.11-2.25)	0.012	1.53 (1.14-2.06)	0.005
Only 1 mental condition (n=213)	1.99 (1.33-2.99)	0.001	1.63 (1.17-2.27)	0.004
Mental conditions multimorbidity (n=59)	3.21 (1.98-5.22)	<0.001	1.99 (1.28-3.11)	0.002
Somatic-mental multimorbidity (n=1542)	3.34 (2.40-4.65)	<0.001	2.36 (1.77-3.16)	<0.001
Urban residence (n=2472)				
No somatic and mental conditions (n=365)	*Reference*		*Reference*	
Only 1 somatic condition (n=459)	0.92 (0.57-1.49)	0.725	0.94 (0.62-1.44)	0.781
Somatic conditions multimorbidity (n=787)	1.59 (1.03-2.44)	0.036	1.33 (0.93-1.91)	0.120
Only 1 mental condition (n=133)	3.32 (2.06-5.35)	0.001	2.20 (1.44-3.36)	<0.001
Mental conditions multimorbidity (n=14)	4.56 (2.02-10.28)	<0.001	3.22 (1.07-9.69)	0.038
Somatic-mental multimorbidity (n=714)	4.57 (3.04-6.88)	<0.001	2.36 (1.61-3.47)	<0.001
Rural residence (n=4256)				
No somatic and mental conditions (n=575)	*Reference*		*Reference*	
Only 1 somatic condition (n=644)	1.53 (1.10-2.11)	0.011	1.48 (1.11-1.99)	0.009
Somatic conditions multimorbidity (n=900)	2.08 (1.57-2.74)	<0.001	1.85 (1.46-2.35)	<0.001
Only 1 mental condition (n=280)	2.09 (1.50-2.93)	<0.001	1.68 (1.28-2.21)	<0.001
Mental conditions multimorbidity (n=70)	3.64 (2.38-5.58)	<0.001	2.07 (1.43-2.98)	<0.001
Somatic-mental multimorbidity (n=1787)	4.27 (3.32-5.48)	<0.001	2.78 (2.23-3.47)	<0.001

*Models adjusted for complex survey design; adjusted for age-sex-education-marital status-smoking status-drinking status-current residence-geographical region-and baseline ADL–IADL index. ADL = activities of daily living; IADL = instrumental activities of daily living; CI = confidence interval.

Stratified analyses by age, sex, and residence are also shown in Table 3. In the adjusted model compared with healthy participants, SMM was associated with a 3.05 (95% CI: 2.29-4.07)-fold increase in ADL-IADL among adults aged 60-69 years, while SMM was associated with a 2.10 (95% CI: 1.55-2.85)-fold increase in ADL-IADL among adults aged 70 years or older. Among males, SMM was associated with a 2.84 (95% CI: 2.15-3.75)-fold increase in ADL-IADL, while SMM was associated with a 2.36 (95% CI: 1.77-3.16)-fold increase in ADL-IADL in females. Among adults who lived in urban areas, SMM was associated with a 2.36 (95% CI: 1.61-3.47)-fold increase in ADL-IADL, while SMM was associated with a 2.36 (95% CI: 2.23-3.47)-fold increase in ADL-IADL among adults who lived in rural areas. The overall *p* values for interaction for the association between multimorbidity combination groups and prospective disability was 0.396 for age, 0.481 for sex, and 0.007 for residence.

In the sensitivity analysis, the association between SMM and the ADL-IADL index did not substantially change after excluding incontinence from the ADL-IADL index (e^β^=2.52, 95% CI: 2.06-3.08) (Supplementary Table 1). The results after further adjustment for body mass index (BMI) were consistent with the results of our main analyses (e^β^=2.52, 95% CI: 2.06-3.08) (Supplementary Table 1). When participants who had at least one impairment in the ADL-IADL index in 2013 were excluded, SMM was associated with a 2.36 (95% CI: 1.77-3.16) times greater ADL-IADL risk in adjusted models (Supplementary Table 2).

## DISCUSSION

This study estimated the prevalence of SMM and investigated the association between SMM and prospective ADL-IADL disability in a nationally representative sample. Overall, we found that 35.7% of Chinese adults aged ≥60 years suffered SMM. Moreover, SMM was independently associated with greater perspective ADL-IADL disability 2 years later when compared with healthy participants free of chronic and mental conditions.

This study demonstrated the clinical pattern of SMM combinations in China, which is the largest aging population in the world. Our findings are consistent with previous studies showing that SMM prevalence was higher in older age groups, females, and those living in rural areas [8, 19, 20, 30, 31]. The prevalence of SMM in our study was higher than that in the Rochester Epidemiology Project of the US population among adults aged ≥ 60 years (35.7% vs. 17.2%), especially among women (44.6% in China vs. 20.4% in the US) [20]. Additionally, we found that older adults who lived in the rural area and Southwest region had a higher rate of SMM compared with those living in other parts of China. This contributes to public health policy and practice to rationally allocate medical resources in China.

Although the prevalence of disability in ADLs and IADLs among older Chinese adults decreased from 1997 to 2006 [32, 33], disability still causes major challenges both for health systems and clinicians in China. Prior studies have shown that chronic multimorbidity is an established risk factor for disability [26–29, 34]. Based on the data from the CHARLS (2013), Qian et al. confirmed that chronic multimorbidity was associated with a 3.66-fold increased risk of disability in ADLs and a 2.00-fold increased risk of disability in IADLs among adults aged ≥ 45 years who had hypertension [35]. However, the contribution of SMM to disability was understudied in this study. To the best of our knowledge, our study was the first to explore the impact of SMM combinations on disability in a nationally representative cohort study in China. The current results support previous findings suggesting that SMM is associated with an increased risk of disability [14, 21, 25, 36]. In the Swedish National Study of Aging and Care in Kungsholmen (SNAC-K) with a 9-year follow-up, SMM was associated with a 0.27 decrease in the number of ADLs per year [21]. Notably, only 4.20% (100 of 2385) of the sample had SMM among older adults ≥ 60 years in SNAC-K study. The statistical power seemed to be low. In addition, in the National Health and Aging Trends Study, older adults with depression and cognitive impairment were associated with a 1.34 times greater risk of disability on the ADL-IADL index [14]. This association was consistent with our findings. This study showed that different patterns of somatic and mental morbidity had different impacts on disability. Given the rapidly developing multimorbidity and disability in the elderly, it is clinically useful to assess the impact of specific multimorbidity combinations on disability.

A major strength of this study was that it was, to the best of our knowledge, the largest prospective nationally representative sample in China to date. Moreover, the methods of data collection were consistent with the Health and Retirement Study and had a higher response rate. However, some limitations need to be addressed. First, chronic conditions/diseases were based on self-report. The self-reporting of somatic conditions may introduce some misclassification bias and underestimate the prevalence of SMM. However, self-report of diagnosed conditions is a common method for determining chronic conditions/diseases in epidemiological studies [14, 25]. In addition, Yuan et al. showed that self-reported diabetes had substantial agreement with fasting glucose and HbA1c levels in the CHARLS [37]. Second, some potential confounding factors need to be considered. We did not adjust for the severity of diseases or the number of medications. Third, 777 participants (11.5%) with complete baseline data were excluded from the study because they were lost to follow-up from 2013 to 2015, potentially causing selection bias. Finally, the long-term trajectories of disability in ADL-IADL were not assessed. Because CHARLS is a relatively new longitudinal cohort, we used the two most recent waves.

In conclusion, our findings show that the patterns of SMM combinations had different effects on disability in older Chinese adults. Moreover, multimorbidity combinations that included depression and/or cognitive impairment were associated with an increased risk of disability.

## MATERIALS AND METHODS

### Study cohort

The current study was based on the China Health and Retirement Longitudinal Study (CHARLS) [38]. A more detailed description of the cohort has been published elsewhere [38, 39]. Briefly, a multistage, stratified and cluster sampling approach was used to obtain this community-based, longitudinal prospective study. Participants were recruited from 6 geographical regions of 28 provinces of China: (1) East (Shanghai, Shandong, Jiangsu, Zhejiang, Fujian, Anhui, and Jiangxi); (2) North (Beijing, Tianjin, Hebei, Shanxi, and Inner Mongolia); (3) Northeast (Liaoning, Jilin, and Heilongjiang); (4) Northwest (Shaanxi, Gansu, Qinghai, and Xinjiang); (5) South-Central (Henan, Hubei, Hunan, Guangdong, and Guangxi); and (6) Southwest (Chongqing, Sichuan, Guizhou, and Yunnan). A computer-aided self-administered questionnaire was used to collect data on sociodemographic characteristics, lifestyle characteristics, health behaviors, chronic diseases, physical function and mental health conditions. The CHARLS national baseline survey was conducted from June 2011 to March 2012 (Wave 1). The above information was repeatedly collected every 2 years. All participants provided written informed consent. This study was approved by the Biomedical Ethics Review Committee of Peking University (IRB00001052-11015).

### Study population

**Figure 2:**
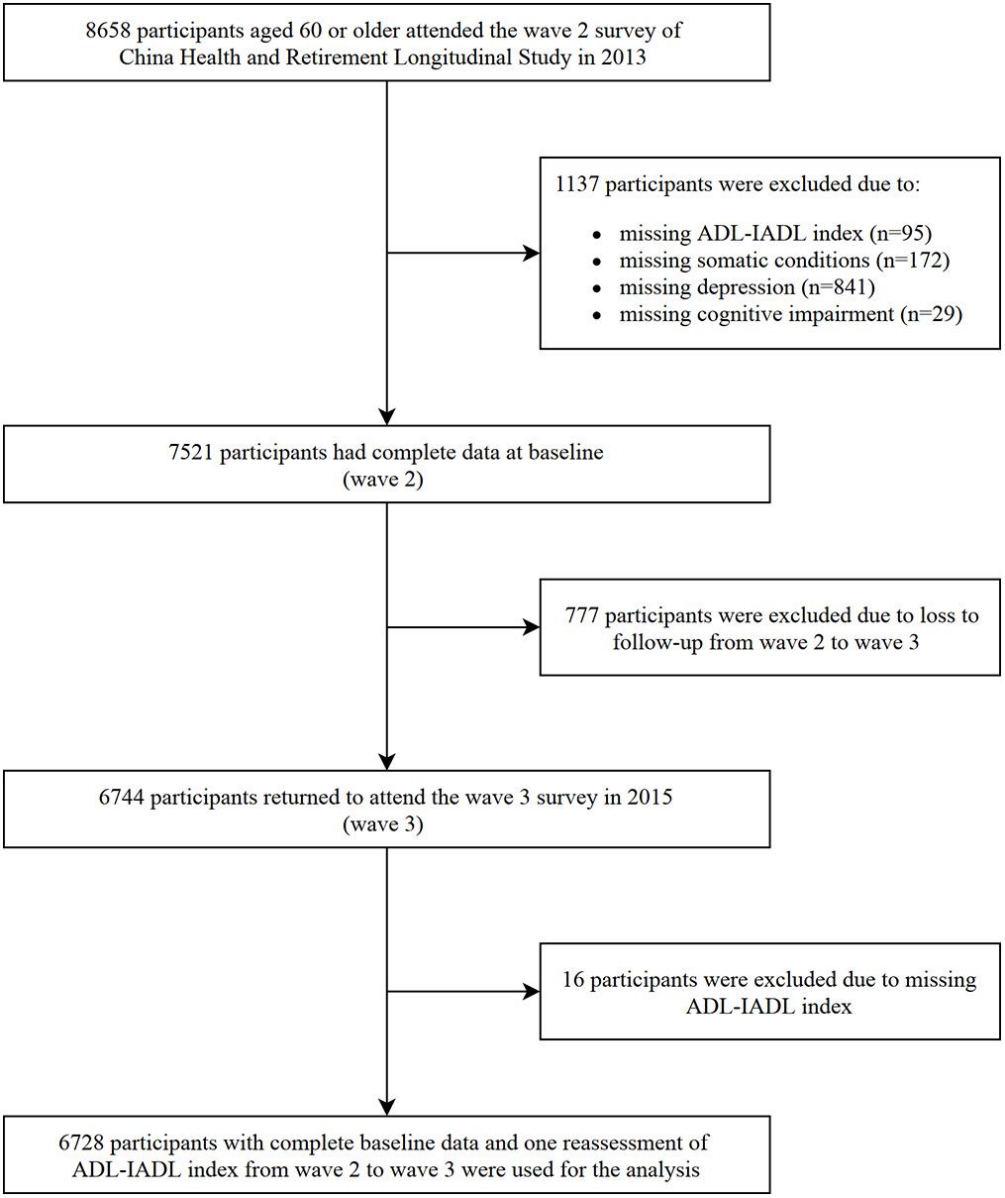
**The age-adjusted somatic-mental multimorbidity (SMM) prevalence by geographic location in China, 2013.**

In Wave 2 (2013), 18612 individuals aged 45 years or over were initially included. The current analysis was limited to 8658 participants who were younger than 60 years of age. We excluded participants who had incomplete data related to the assessment of chronic diseases, depression, cognitive function, disability at baseline, follow-up or loss to follow-up in Wave 3 (2015). Finally, a sample of 6728 participants was included in the current analysis. A flowchart of the selected participants is shown in Figure 2.

### Disability assessment

Disability was assessed using a modified version of the Katz Index of Independence in Activities of Daily Living (ADL) [40] and the Lawton Instrumental Activities of Daily Living (IADL) Scale [41]. Participants were asked whether they had difficulty or needed help performing 6 ADLs (dressing, bathing, eating, getting in and out of bed, toileting, and incontinence) and 5 IADLs (managing money, taking medications, grocery shopping, preparing meals, and making phone calls). Both ADLs and IADL were assessed at baseline (Wave 2, 2013) and follow-up (Wave 3, 2015). The ADL-IADL index was calculated by summing the number of impairment activities in ADL and IADL for each participant who had at least one no missing item on the ADL and IADL instruments (range: 0-11) [14]. A higher ADL-IADL index represented a greater disability severity.

### Somatic conditions/diseases

Self-reported physician-diagnosed diseases in 2013 or prior prompted by “Has a doctor ever told you that you have…,” included hypertension, diabetes, cancer or a malignant tumor (excluding minor skin cancers), chronic lung disease, heart disease (heart attack, coronary heart disease, angina, congestive heart failure, or other heart problems), stroke, arthritis, dyslipidemia (elevation of low density lipoprotein, triglycerides, and total cholesterol, or a low high density lipoprotein level), liver disease (except fatty liver, tumors, and cancer), kidney disease (except for tumor or cancer), digestive disease (except for tumor or cancer), and asthma.

### Mental health conditions

Mental health conditions, including depression and cognitive impairment, were assessed in 2013.

### Depression

We used two types of information to identify whether participants were depressed in 2013: (1) self-report of physician-diagnosed psychiatric problems and (2) the 10-item Center for Epidemiological Studies Depression Scale (CESD-10) [42]. The CESD-10 scores were calculated by summing the responses to each question: 0 = rarely or none of the time (less than 1 day), 1 = some or a little of the time (1-2 days), 2 = occasionally or a moderate amount of time (3-4 days), 3 = most or all of the time (5-7 days). The CESD-10 scores ranged from 0 to 30, with higher scores indicating that the participant felt more negatively during the past week. As suggested by a previous study [43], a score of ≥ 10 on the CESD-10 indicated a positive screen for depressive symptoms in older Chinese adults. Depression was defined as having either self-reported psychiatric problems or a CESD-10 score ≥ 10.

### Cognitive impairment

Cognitive impairment was defined as having either self-reported dementia or Alzheimer’s disease diagnosed by a doctor or impairment in at least 2 cognitive tests. Three tests were administered: (1) the Telephone Interview of Cognitive Status (TICS-10) (range: 0-10); (2) word recall (range: 0-10); and (3) figure drawing (yes=1, no=0). A detailed description of the cognitive assessments used in the CHARLS was published previously [44, 45]. Impairment on each cognitive test was defined as scores at or below 1.5 standard deviations (SD) from the mean for the CHARLS study sample. Score cutoff points for ≤ 1.5 SD below the mean on each cognitive test are shown in Supplementary Table 2.

### Definition of multimorbidity

Following previous studies [20, 25], we defined general multimorbidity as the presence of 2 or more of the 14 conditions in CHALRS 2013. Similarly, we defined SMM as any combination of two or more conditions in which at least one condition was somatic and at least one condition was mental.

### Covariates

Sociodemographic characteristics included age, sex, education (less than lower secondary, upper secondary, and vocational training, and tertiary), marital status (married and others: widowed, separated, divorced and never married), current residence (urban and rural area), and geographical region. Lifestyle variables included self-reported smoking and drinking status (never, ever, and current). BMI was calculated as weight in kilograms divided by height in meters squared. The baseline ADL-IADL index indicated the severity of disability reported in 2013.

### Statistical analysis

All analyses were performed using Stata 15 software (College Station, TX). A *p*-value <0.05 was considered statistically significant. Descriptive statistics were presented as the mean (standard deviation) for continuous variables and percentage for categorical variables.

We first estimated the sex-specific prevalence of SMM in the overall population and then according to age group (60-64, 65-69, 70-74, 75+ years) and current residence. The χ^2^ test was used to compare the crude prevalence. We also identified the prevalence of SMM by geographical region, adjusting for age as a continuous variable in logistic regression. We used the “svy: tabulate” and “svy: logit” procedures in Stata to estimate the prevalence while taking the complex survey design and the nonresponse rate of the CHARLS survey into account.

In order to assess the impact of different combinations of SMM in 2013 and ADL-IADL disability in 2015, the combinations identified in 2013 are presented in Supplementary Figure 1. As the outcome variable was a count variable, a negative binomial regression model (NBRM) was fitted for complex survey design and overdispersion to investigate the impact of multimorbidity combinations in 2013 on the ADL-IADL index in 2015. NBRM was conducted using the “svy: nbreg” procedure in Stata. Two models were built. Model 1 was unadjusted, and Model 2 was adjusted for age (continuous), sex, education, marital status, smoking status, drinking status, current residence, geographical region, and baseline ADL-IADL index. We reported the exponentiated regression coefficients and corresponding 95% CIs, representing the difference in the number of ADL-IADL impairments between each multimorbidity combination group compared to healthy participants (reference).

A stratified analysis was performed by age group (< 70 and ≥ 70 years), sex (male and female), and current residence (urban and rural area). The cutoff age of 70 years was chosen because it was close to the average age of the study population (mean: 68 years). Interaction terms between multimorbidity combination groups and the above subgroups were added in the multivariable model, and a *p-*value for interaction was obtained from the likelihood test by comparing models with and without the interaction term.

Three sensitivity analyses were conducted: (1) because incontinence is more likely to be considered an organic disorder rather than a disability in clinical practice [46], we recalculated the ADL-IADL index (range: 0-10) by removing incontinence; (2) we additionally adjusted for BMI (continuous) in the subpopulation with complete data for BMI (n=5320); and (3) we excluded participants who had at least one impairment in the ADL-IADL index in 2013 (n=4061).

## Supplementary Material

Supplementary Figure 1

Supplementary Tables
