# Supplementary material for: Prevalence of somatic-mental multimorbidity and its prospective association with disability among older adults in China
Source: Aging (Albany NY). 2020 Apr 25;12(8):7218–31. doi: 10.18632/aging.103070 (PMC7202546; doi:10.18632/aging.103070)
Supplement: Supplementary Figure 1 [file aging-12-103070-s001..pdf]

SUPPLEMENTARY FIGURE

No. Mental conditions (MC)	No. Somatic conditions/diseases (SC)		
	0	1	2+
0	No	1 SC	SC Mult.
1	1 MC		
2	MC Mult.		SMM

Supplementary Figure 1. Model showing the grouping of somatic and mental conditions/diseases. Participants were grouped by the number of somatic conditions (SC) and mental conditions (MC). Mult., multimorbidity; Ref., reference group; SMM, somatic-mental multimorbidity.
