# Supplementary material for: Prevalence of somatic-mental multimorbidity and its prospective association with disability among older adults in China
Source: Aging (Albany NY). 2020 Apr 25;12(8):7218–31. doi: 10.18632/aging.103070 (PMC7202546; doi:10.18632/aging.103070)
Supplement: Supplementary Tables [file aging-12-103070-s002..pdf]

SUPPLEMENTARY TABLES

Supplementary Table 1. Sensitivity Analyses of Association Between Combination of Somatic and Mental Conditions and ADL–IADL Index.

Multimorbidity combination groups	Adjusted* e^{β} (95% CI)	*p* value
Sensitivity analyses 1: removing incontinence from ADL-IADL index (n=6728)		
No somatic and mental conditions (n=940)	*Reference*	
Only 1 somatic condition (n=1103)	1.25 (0.98-1.61)	0.076
Somatic conditions multimorbidity (n=1687)	1.56 (1.27-1.93)	<0.001
Only 1 mental condition (n=413)	1.86 (1.45-2.39)	<0.001
Mental conditions multimorbidity (n=84)	2.04 (1.39-3.00)	<0.001
Somatic-mental multimorbidity (n=2501)	2.52 (2.06-3.08)	<0.001
Sensitivity analyses 2: additionally adjusted for body mass index (continuous) (n=5320)		
No somatic and mental conditions (n=756)	*Reference*	
Only 1 somatic condition (n=873)	1.14 (0.85-1.53)	0.373
Somatic conditions multimorbidity (n=1339)	1.56 (1.22-2.00)	<0.001
Only 1 mental condition (n=326)	1.73 (1.31-2.29)	<0.001
Mental conditions multimorbidity (n=66)	1.85 (1.21-2.81)	0.004
Somatic-mental multimorbidity (n=1960)	2.57 (2.04-3.25)	<0.001
Sensitivity analyses 3: excluding participants who had at least one impairment in ADL-IADL index in 2013 (n=4061)		
No somatic and mental conditions (n=772)	*Reference*	
Only 1 somatic condition (n=818)	1.12 (0.79-1.59)	0.532
Somatic conditions multimorbidity (n=1147)	1.38 (1.03-1.85)	0.031
Only 1 mental condition (n=239)	1.92 (1.36-2.73)	<0.001
Mental conditions multimorbidity (n=30)	2.20 (0.95-5.11)	0.065
Somatic-mental multimorbidity (n=1055)	2.99 (2.27-3.95)	<0.001

*Models adjusted for complex survey design; adjusted for age-sex-education-marital status-smoking status-drinking status-current residence-geographical region-and baseline ADL–IADL index. ADL = activities of daily living; IADL = instrumental activities of daily living; CI = confidence interval.

Supplementary Table 2. Score cutpoints for ≤1.5 SDs below mean on cognitive tests.

	TICS-10	Word-recall	Figure-drawing
Score range	0-10	0-10	0-1
Score mean	5.95	3.26	0.17
Score SD	3.10	1.79	0.38
Score cutpoints	≤1	≤1	0
